# Depersonalisation-derealisation as a transdiagnostic treatment target: a scoping review of the evidence in anxiety, depression, and psychosis

**DOI:** 10.3389/fpsyg.2025.1531633

**Published:** 2025-04-16

**Authors:** Emma Černis, Milan Antonović, Roya Kamvar, Joe Perkins, The Transdiagnostic DPDR Project Lived Experience Advisory Panel, Louise Chandler, L. Corrigan, Nanette Lee, Sara Metz, Judah Njoroge

**Affiliations:** ^1^School of Psychology, University of Birmingham, Birmingham, United Kingdom; ^2^The McPin Foundation, London, United Kingdom; ^3^Independent Researcher, Bristol, United Kingdom

**Keywords:** anxiety, depersonalization, depression, derealization, dissociation, intervention, psychosis, treatment

## Abstract

**Introduction:**

Depersonalisation and derealisation (DPDR) describe dissociative experiences involving distressing feelings of disconnection from oneself or one’s surroundings. Such experiences are common transdiagnostically across the range of mental health presentations, with evidence to suggest they may even play an active role in the development and maintenance of other mental health concerns. If substantiated, DPDR could present a plausible novel transdiagnostic treatment target. The objective of this scoping review was to therefore to synthesise the evidence-base regarding DPDR as a transdiagnostic target for the treatment of anxiety, depression, and psychosis, in order to evaluate this proposal for each.

**Methods:**

Embase, Ovid MEDLINE, APA PsychInfo, Scopus, and PubMed were searched for empirical published research and “grey” literature addressing transdiagnostic DPDR and primary anxiety, depression, or psychotic disorders (time range: 1993 to 12th October 2023). Extracted data were summarised and provided to the Lived Experience Advisory Panel for interpretation and analysis.

**Results:**

We screened 3,740 records, resulting in 42 studies addressing DPDR in the context of psychosis, 28 in anxiety, and 24 in depression. The results indicate that transdiagnostic DPDR is highly likely to be a viable treatment target in psychosis, and that it may share common cognitive processes with anxiety disorders. Evidence for the feasibility of DPDR as a treatment target in depression was sparse, and thus inconclusive.

**Discussion:**

Whilst no established interventions targeting transdiagnostic DPDR were identified by this review, its findings highlight many viable options for treatment development. Given the difficulty drawing clinically meaningful conclusions from the current evidence-base, we strongly recommend that this work actively involves people with lived experience of DPDR.

**Systematic review registration:**

https://osf.io/ufbkn/.

## Highlights

- Transdiagnostic DPDR is likely to be a viable treatment target in psychosis.
- Transdiagnostic DPDR may share modifiable common cognitive processes with anxiety.
- The relationship between DPDR and depressive disorders is under-researched.
- No established treatment options exist for DPDR in any of these three contexts.

## 1 Introduction

Dissociative symptomatology encompasses a broad range of experiences, with a multidimensional framework suggesting various approaches to subdividing these experiences into helpful categories (for example, compartmentalisation and detachment) (Holmes et al., 2005). Depersonalisation and derealisation (DPDR) are established subtypes of dissociative experience involving feelings of disconnection from oneself, including one’s body, thoughts, or feelings (depersonalisation), or from one’s surroundings, including the environment and people around you (derealisation) (Kennedy et al., 2013). Difficult to describe, the phenomenology of DPDR is highly subjective (e.g., “it’s as though there’s a pane of glass between me and the world”; “I know I was there, but it feels like someone else’s memory”). Such experiences may be transient and benign, causing little to no distress. However, in cases of acute or chronic DPDR, a diagnosis of Depersonalisation-Derealisation Syndrome (World Health Organization, 2022) or Depersonalization/Derealization Disorder (American Psychiatric Association, 2013) may be made.

DPDR experiences are common in the general population and highly prevalent across mental health disorders (Lyssenko et al., 2018), including in anxiety (at a rate of up to 20.2%), depression (approximately 50%), and psychosis (up to 50%) (Renard et al., 2017; Yang et al., 2023). As a facet of dissociation, it may thus be considered transdiagnostic (occurring across a range of mental health presentations, cross-cutting diagnostic boundaries) (Ellickson-Larew et al., 2020), and likely to play a central role in the broader landscape of mental health (Černis et al., 2021).

Given its high prevalence transdiagnostically, and since dissociative experiences such as DPDR have been linked to important clinical outcomes, such as treatment response (Bae et al., 2016), suicidal ideation (Pachkowski and Klonsky, 2023), and risk of self-harm and suicide attempts (Černis et al., 2019; Foote et al., 2008), it is important to consider whether DPDR may constitute a novel treatment target in interventions for a range of mental health difficulties. That is, whether DPDR may be acting as a causal or maintaining process in disorders such as anxiety, depression, and psychosis, and thus whether alleviating DPDR may consequently reduce these, too.

Recent reviews of plausible mechanisms of the broader construct of dissociation (Lynn et al., 2019; Lynn et al., 2022) have identified modifiable factors that could feasibly be similar across diagnoses (e.g., emotion dysregulation). This is important, since treatments for DPDR could be developed by considering how to effectively target these specific mechanisms – for example, as has been achieved in recent interventionist-causal work in the field of paranoia (Freeman, 2016; Freeman et al., 2016). However, these reviews have not specifically addressed DPDR, and thus have not determined whether the mechanisms of DPDR are the same across diagnoses. This would therefore be an important question to address early in the development of any transdiagnostic intervention for DPDR.

However, despite their potential, the treatment of dissociative experiences is a significant area of unmet need in mental healthcare (Sar, 2011), and the evidence-base for treatment of dissociation is still in infancy. At the time of writing, a Depersonalisation-Derealisation Disorder-specific CBT feasibility study is currently underway in London, UK (ISRCTN40944), but no other evidence-based interventions for DPDR exist – either as a discrete diagnosis in its own right, or as a transdiagnostic phenomenon in the context of another disorder. As such, there is no National Institute for Health and Care Excellence (NICE; UK) or National Institutes of Health (NIH; United States) clinical guidance for the treatment of DPDR.

Such treatment is desperately needed. Qualitative evidence (Černis et al., 2020) and testimonials from lived experience experts (Perkins, 2021) clearly illustrate that DPDR leads to diminished quality of life due to withdrawal from social activities, relationships, interests and hobbies, and due to difficulties with day-to-day functioning. There is a growing service user movement regarding DPDR, with the lack of recognition in routine clinical services (Brand, 2016) and unacceptable length of time to diagnosis of Depersonalization-Derealization Disorder (Hunter et al., 2017) raised in the House of Commons in the UK (HC Deb 12 March 2019). Indeed, the desire to address DPDR is such that a specialist charity – ‘Unreal’ – was launched in the UK in 2020 to increase awareness and provide peer support. The charity is now overwhelmed with requests for support and advice (J. Perkins, *personal communication, May 2022*).

Given this context, an overview of what is known about the treatments and mechanisms of DPDR as a possible transdiagnostic treatment target would be timely and a highly valuable addition to the literature.

## 2 Aims

Thus, the objective of this scoping review was to synthesise the evidence-base regarding DPDR as a transdiagnostic target for depression, anxiety, and psychosis, and to understand the extent and type of knowledge currently available, with a view to informing future clinical guidance and treatment development efforts. Specifically, we sought to address the following research questions:

1) What is the state of the evidence-base for DPDR as a transdiagnostic target for anxiety, depression, and psychosis?
2) Do any treatments already exist for DPDR in the context of anxiety, depression, or psychosis?
3) What are the plausible mechanisms of action for DPDR in these contexts (that could be the focus of treatment development in future)?

## 3 Method

We took a systematic scoping review approach (Peters et al., 2015), and pre-registered our search protocol on the Open Science Framework (https://osf.io/ufbkn/).

### 3.1 Search strategy

#### 3.1.1 Piloting

After a search of PROSPERO revealed no similar systematic reviews underway, we piloted and refined our search strategy via an initial search using Embase (only) on 28th March 2023. Two reviewers (EČ and MA) rated the first 77 results (i.e., all results where the first authors’ surnames began with ‘A’), to develop inter-rater agreement regarding the application of the eligibility criteria and to establish inter-rater reliability.

Following piloting, it was agreed that: the final searches would be limited to the past 30 years only, and that acronyms (e.g., “DPD” for Depersonalisation-Derealisation Disorder) would be omitted from the search terms for subsequent searches, as older research and acronyms raised many irrelevant results. Additionally, it was agreed that that Embase, Ovid MEDLINE, APA PsychInfo, Scopus, and PubMed would all be searched, as search results between databases were not identical.

#### 3.1.2 Final searches

All three searches used the following terms: “depersonalisation OR depersonalization OR derealisation OR derealization NOT burnout”.

The psychosis search (only) additionally used: ‘OR DPD OR DPDR OR ‘DP/DR’ OR DPAFU OR ‘DP-DR’ OR DPRD’ AND ‘psychosis OR schizophreni* OR schizoaffective OR psychotic* Or hallucinat* OR delusion* OR paranoi* OR grandios* OR ‘first rank symptom*”.

The anxiety search additionally used: ‘AND anxiety OR *phobia OR phobi* OR ‘obsessive compulsive disorder’ OR OCD OR ‘panic disorder’ OR panic’.

The depression search additionally used: ‘AND depress* NOT bipolar’.

‘NOT burnout’ was specified since ‘depersonalisation’ is a term often used in relation to workplace stress and employee burnout, but in this context relates to objectifying or de-humanising another person (as opposed to feeling unreal or disconnected about oneself).

The psychosis search took place on the 28th March 2023, with automated search updates included up until the 17th October 2023 (inclusive). The anxiety search took place on 25th May 2023, and the depression search on 2nd August 2023. Automated search updates for these were included in the results until 12th October 2023.

### 3.2 Inclusion criteria

Any literature identified by the above searches that presented novel empirical data pertaining to the relationship between DPDR and anxiety, depression, or psychosis in humans was eligible for inclusion.

#### 3.2.1 Types of evidence

Here, ‘novel’ and ‘empirical’ indicate that expert opinion, narrative reviews of evidence, and descriptions of clinical trends or phenomena (without measurement) would not be eligible for inclusion. Systematic reviews and meta-analyses were searched for relevant references but were themselves only included if they presented novel empirical analysis.

Unpublished or ‘grey’ literature – e.g., conference abstracts, letters to the editor, PhD theses – were eligible for inclusion in this review. Additionally, experts and early career researchers in the field were contacted by EČ to explain the aim and scope of the review and request access to any relevant unpublished literature.

Results where the main finding was a confirmation that DPDR is transdiagnostically associated with anxiety, depression, and psychosis (i.e., findings of a statistically significant correlation, or that rates of DPDR are high in these diagnostic groups) were not included.

#### 3.2.2 Core concepts

To address the above research questions, only results where DPDR and also (one or more of) depression, anxiety, or psychosis are primary or secondary factors of interest were included. These constructs had to be considered in direct or indirect relation to each other. Therefore, research addressing possible mediators between DPDR and anxiety, depression, or psychosis; or those proposing a mechanism of DPDR within these specific contexts, were included. However, articles that discuss DPDR alone, as a side-effect of medication or substance intoxication (only), or studies where DPDR was never analysed in relation to the other construct, were excluded. Studies where a mechanistic relationship was implied, but not tested for were also excluded (e.g., findings of a correlation between a third factor and transdiagnostic DPDR in the context of anxiety, depression, or psychosis, without mediation testing).

Consistent with the conceptualisation of DPDR as a transdiagnostic phenomenon, DPDR was conceptualised as a dimensional variable (i.e., a state or trait latent construct) that could occur in any diagnostic context, whereas anxiety, depression, and psychosis were only considered in terms of diagnostic phenomena. Where conceptualisation of anxiety, depression, or psychosis was unclear, the context of the study was considered and results based on measurement of the construct using clinically-relevant (e.g., symptom count) measures were more likely to be included than those adopting trait measures.

To reduce the scope of the review and reduce the complexity of the key constructs, anxiety, depression, and psychosis were limited to include only diagnostic entities from these three chapters of diagnostic manuals, and where these were the primary presenting difficulties. More specifically, ‘anxiety’ included anxiety disorders where anxiety is the primary difficulty (e.g., OCD, social phobia, etc.) – but associated phenomena (e.g., worry, insomnia), non-clinical presentations (e.g., “anxiety proneness”), and anxiety-related diagnoses (e.g., body-focused repetitive behaviors) were not considered to fulfil this criterion. ‘Depression’ included depressive disorders where depression is the primary difficulty – but associated phenomena (only) (e.g., suicidality; wellbeing), strictly non-clinical presentations (e.g., “low mood”), and psychotic depression were excluded. Similarly, the inclusion criteria for the psychosis search were: any non-organic non-affective psychotic disorder – i.e., disorders where the primary difficulty is psychotic experience, such as those listed in the DSM-5 ‘Schizophrenia Spectrum and Other Psychotic Disorders’ chapter. Thus, this search included schizophrenia, but excluded disorders where psychosis may be associated, but is not the primary presenting problem (e.g., bipolar disorder, schizotypal personality disorder). Research concerning psychotic symptoms (e.g., paranoia, delusions, and hallucinations) in isolation were considered for inclusion. However, neurological, or organic psychosis/psychotic symptoms (e.g., hallucinations arising in Parkinson’s Disease, or in the context of migraine) were excluded.

#### 3.2.3 Procedure

The search results from all five databases were imported into Zotero version 6.0.26 (Zotero, 2023) and duplicates removed.

Given the high inter-rater agreement in the pilot testing, one reviewer (MA) screened the title and abstracts of the results according to the eligibility criteria and flagged results requiring full-text review via the tagging function within the software.

Concurrently, a second reviewer (EČ) second-rated a random selection of excluded results, and carried out a full-text review of flagged results. Uncertainty regarding the eligibility of a search result was resolved via discussion between the research team (EČ, MA, RK and JP) or by bringing the result to an analysis meeting with the Lived Experience Advisory Panel (LEAP). Where necessary, authors of papers were contacted to request missing or additional information.

During full-text review, data from eligible studies were extracted by EČ, according to the preregistered protocol^1^, and recorded in a Microsoft Excel file. No critical appraisal or quality of evidence scoring tools were used, since these are not required in scoping reviews (Arksey and O’Malley, 2005), although the type of study was extracted as this allows comment on the level of evidence, relevant to the first research question.

Results were divided into ‘treatment’ and ‘mechanistic’ studies, for the purposes of answering research questions two and three, respectively. Studies were considered ‘treatment’ if they concerned the outcome of an intervention to ameliorate transdiagnostic DPDR in the context of the major diagnosis (anxiety, depression, psychosis); or where an effect on DPDR was noted as a result of an intervention intended to treat anxiety, depression or psychosis. A result was considered a ‘mechanistic’ study if its findings offered further insight into the causal relationship between DPDR and the diagnosis in question; or where a separate factor was implicated in the development or maintenance of, or relationship between DPDR and anxiety, depression, or psychosis.

### 3.3 Lived experience involvement

Involvement of people with lived experience of DPDR was integral to this review. Every stage of this project – the design, management, review, and delivery – involved people with lived experience of DPDR. This was achieved through three strands of the project: the core research team, the LEAP, and in the dissemination of results.

In the core research team, two of the authors have long-standing lived experience of DPDR and were involved in the design of the project, the application for funding, and the overall direction of progress throughout. Monthly meetings with the core team were held to ensure all key decisions were made together, and thus were shaped by lived experience perspectives.

The LEAP was recruited by advertising via the McPin Foundation (a UK-based charity for lived experience inclusion in mental health research) and the Mental Health Innovation Network (which is jointly led by the Centre for Global Mental Health at the London School of Hygiene & Tropical Medicine, and the World Health Organisation). Applications were reviewed by the two members of the core team with lived experience of DPDR, and six LEAP members chosen reflecting a diversity of experience across DPDR, anxiety, depression, and psychosis. One LEAP member declined to participate after the first meeting, resulting in a panel of five. The majority of LEAP members identified as female and most were based in the UK, with one member based in Kenya. Four LEAP meetings were held: one to orient members to the project, and three to interpret the psychosis, anxiety, and depression search findings (see *Analysis*). Additionally, regular contact was maintained with LEAP members via email or video progress updates, and through answering queries on an online discussion board (where contributions could be named or anonymous).

Finally, the plain English summary of results was written by a member of the core research team with lived experience of DPDR and edited by the LEAP, and the video and infographics were produced by suppliers with lived experience of DPDR (Supplementary material).

### 3.4 Analysis

As requested by the LEAP, simplified versions of the extracted data tables were provided a week prior to each of the three analysis meetings. Tables were simplified by omitting columns containing superfluous or technical detail (e.g., the names of the measures), providing concise plain English explanations of the study findings, and appending a glossary of important terms and concepts (e.g., statistical mediation).

In each analysis meeting, the LEAP iteratively interpreted the data by discussing the themes and patterns observed in the data table. They were supported by RK and JP to draw their own conclusions about the key findings, and highlight any surprises or notable omissions. EČ was present throughout these discussions to provide further detail or answer any questions about the studies or terms included in the table, if requested.

Following the analysis meetings, the LEAP’s discussion was synthesised into a narrative and used as the basis for the review results.

## 4 Results

**Figure 1:**
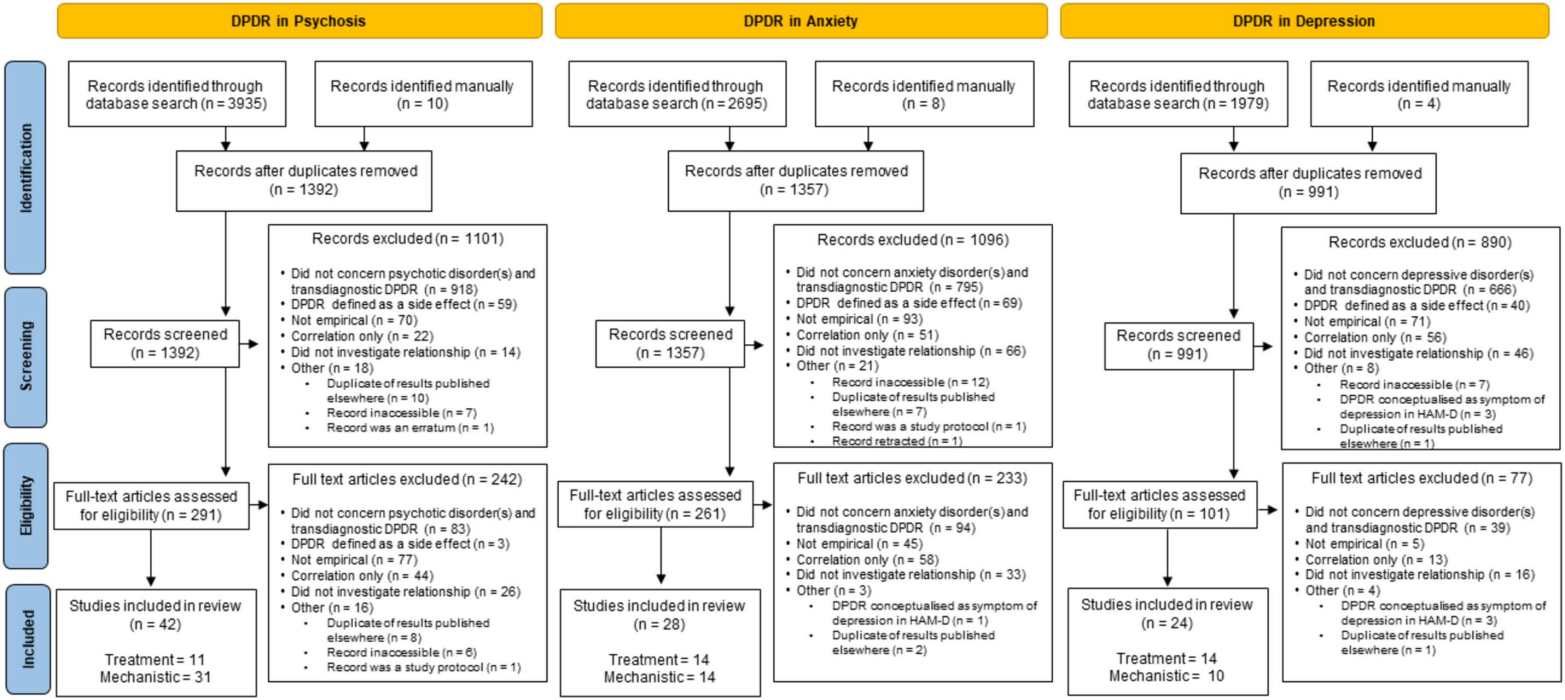
The PRISMA (Tricco et al., 2018) diagram for all three searches included in this scoping review. “Duplicate of results published elsewhere” includes conference presentations or theses excluded in favour of the subsequently published peer-reviewed article, or findings that were published in multiple journals. DPDR = transdiagnostic depersonalisation-derealization. HAM-D = Hamilton Depression Rating Scale (Hamilton, 1960).

Searches identified 42 relevant studies addressing DPDR in the context of psychosis, 28 in anxiety, and 24 in depression (Figure 1). These results came from 76 papers, theses, and conference reports (Supplementary material).

### 4.1 DPDR in psychosis

#### 4.1.1 What is the state of the evidence-base for DPDR as a transdiagnostic target for psychosis?

As illustrated in Figure 1, of the 1,392 results from the psychosis literature search, the majority (*n* = 1,001) did not concern DPDR experiences in the context of psychosis, and a further 62 only conceptualised DPDR as a side effect of medication. Many (*n* = 147) approached the topic in a narrative or otherwise non-empirical way. Of those that did take an empirical approach to measuring transdiagnostic DPDR in the context of psychosis, 40 did not test the relationship between the two constructs, and 66 merely demonstrated a correlation between DPDR and psychosis, without expanding further on the nature or direction of the association.

Forty-two studies did address the relationship in sufficient depth for us to consider them in relation to our second and third research questions: 11 reported on treatment efforts in this context (Table 1), and 31 (Table 2) were considered as mechanistic studies, as outlined in *Procedure*.

**Table 1:** Summarising the treatment studies addressing the second research question for all three searches (DPDR in anxiety, depression, and psychosis).

Author	Year	Type of study	Treatment	Dose/duration	Context/diagnosis	*N*	Mean age	Key finding
Psychosis								
Eyoum et al.	2021	Case study	Antipsychotics (not stated), supportive psychotherapy & psychomotor techniques.	2–6 weeks of treatment.	Schizophrenia	2	26 & 27	Treatment improved muscle tone, general coordination, and drawings of themselves, implying better connection to the body/self. Psychotic symptoms not re-assessed. 6-month follow-up results not stated.
Farrelly et al.	2023	Single-blind feasibility RCT	CBT (vs. TAU)	6x weekly 1-h sessions.	Adults with active psychotic symptoms	21	40.5	Reduction in DP and hallucinations. Two thirds of intervention group no longer met criteria for DPD, versus one third of control group. Treatment feasible and had highly acceptability.
Morikawa et al.	1998	Case study	Bromocriptine	15 mg daily, in addition to bromperidol.	Schizophrenia with pituitary gigantism	1	30	Psychotic symptoms improved with bromperidol alone, but DP did not. Adding bromocriptine 7.5 mg reduced DP, and 15 mg resolved it completely.
Narita et al.	2018	Case study	ECT	12 sessions bilaterally; in addition to quetiapine, olanzapine & asenapine.	Psychosis in the context of multiple sclerosis	1	42	Whilst the medication and ECT improved auditory hallucinations, they had no effect on DP.
Piedfort-Marin	2019	Case study	Hypnotherapy	14 sessions	Postpartum psychosis	1	35	After 4 sessions the client had no psychotic or DP symptoms. Remained symptom-free at follow-up 2.5 years after.
Romain et al.	1996	Open study	Zuclopenthixol acetate (injections)	Most patients: 126-138 mg, 3 doses, each 3 days apart. Some received higher doses.	Psychotic disorders	46	32	Whilst ‘psychotic anxiety’ reduced, DP did not.
Rault et al.	2022	Open study	VR relaxation	5× 30-min sessions	Schizophrenia & Schizoaffective disorder	13	43	DP score significantly reduced, even after one session. Post-VR psychotic symptoms not assessed.
Richa et al.	2009	Case study	Amisulpride	400 mg per day	Paranoid Schizophrenia + Niemann-Pick disease type B	1	27	Delusions, auditory hallucinations and DPDR ‘regressed’ and the patient was able to return to work. Follow up maintained for more than 10 years.
Yoshimura et al.^+^	2020	Case study	Brexpiprazole	2 mg per day	Schizophrenia	1	41	DP “improved” after 28 days. Effect on psychotic symptoms not explicitly stated but patient described as “well.”
Anxiety								
Mavissakalian	1996	Double-blind RCT	Imipramine vs. placebo or low dose	Medium (1.5 mg/kg/day) or high dose (3 mg/kg/day) vs. placebo or low dose (0.5 mg/kg/day)	Outpatients with panic disorder and agoraphobia.	80	36.4	‘Unreality’, as a symptom of panic disorder improved to a greater extent in the med-high group than in the placebo-low group from Week 0 to Week 4 and from Week 4 to Week 8.
McKay and Moretz	2008	Case series	Interoceptive exposure for DPDR (using 3D glasses)	1-3× 50-min exposure session, with 30-min daily practice at home.	Outpatients with panic disorder and agoraphobia undergoing CBT for panic who reported significant DP during panic attacks.	3	31.7	Two of the three patients reported significant decreases in DP during panic attacks after one exposure session. All three showed reductions in measures of panic & agoraphobia post-treatment.
Pegna et al.	1999	Case study	Citalopram	20 mg per day	Panic disorder.	1	12	Complete remission of panic and DPDR symptoms after a month of treatment. However, ‘she still sometimes experienced brief episodes of [DPDR] without fear’.
Preve et al.^+^	2013	Case study	Ziprasidone	40 mg per day (plus 40 mg paroxetine & 50 mg lamotrigine)	Outpatient with panic disorder and DPDR (& comorbid bipolar disorder).	1	35	More than 50% improvement in DP score once ziprasidone was introduced—the other two medications improved depressive and panic symptoms, but not DPDR.
Schweden et al.	2016	RCT	Cognitive therapy for social anxiety vs. waitlist control	Up to 25 sessions.	Outpatients with a diagnosis of social anxiety disorder.	40	25.3	Participants who showed a ‘therapy response’ in their social anxiety showed the biggest declines in DPDR scores during Trier Social Stress Test.
Simeon et al.	2004	Double-blind RCT	Fluoxetine vs. placebo	10-60 mg per day.	Adults with DPD (*n* = 9/25 in treatment arm had a comorbid anxiety disorder)	50	35.7	Fluoxetine did not improve DPDR, but where it improved comorbid anxiety (*n* = 4) all showed improvements in DPDR. Where anxiety did not improve (*n* = 5), only one had improvements in DPDR.
Uguz and Sahingoz	2014	Case study	Aripiprazole	10 mg per day (plus 20 mg escitalopram).	Outpatient with OCD and DPD.	1	25	Only aripiprazole improved DPDR. This happened ‘dramatically’ within 2 weeks. Benefits maintained at 8 week follow-up.
Weber	2020	Case study	Mixed amphetamine salts	20 mg per day (plus 225 mg venlafaxine & psychotherapy).	Generalised anxiety disorder and DPD.	1	35	Venlafaxine improved anxiety but not DPDR. With mixed amphetamine salts, ‘[DPDR] symptoms did not fully resolve, [but] the patient reported a notable reduction in the frequency and intensity of her symptoms as well as improved functioning and quality of life’.
Depression								
Rotaru et al.	2015	Case study	CBT-informed psychotherapy	10 sessions.	MDD with suspected psychotic features.	1	21	DPDR was a key complaint. Six months after therapy, still had DR episodes ‘but they did not scare him as much as they did in the past’.
Uguz and Sahingoz	2014	Case study	Aripiprazole	10 mg per day (plus 150 mg venlafaxine).	Female outpatient with depression and DPDR.	1	36	DPDR only resolved after initiation of aripiprazole. Patient reported a ‘complete improvement’ at 6 weeks. Depression resolved.
				10 mg per day (plus 40 mg paroxetine).	Female inpatient with depression and DPD.	1	38	DPDR ‘almost completely recovered’ after 6 weeks. Depression fully remitted.
Belli et al.^+^	2014	Case study	Sertraline & lamotrigine	Sertraline 200 mg per day, lamotrigine 100 mg.	Female outpatient with chronic depression and DP.	1	37	Marked improvement in DPDR and depression after 10 days. Euthymic after 8 weeks (DPDR not reported). Maintained at 6-month follow-up.
Karris et al.	2017	Case study	rTMS	24 sessions 1 Hz/s for a total of 1,200 pulses over 20 min; then 8 sessions 10 Hz over 4 s with 26 s quiet interval, for total of 3,000 pulses over 37.5 min (plus buproprion).	Male outpatient with MDD and DPD.	1	30	First 24 rTMS sessions gave “gradual improvement” in depression and DPDR. Patient requested higher frequency and after this, “noted obvious improvements” in DPDR. Remains stable with maintenance treatment.
Ordas and Ritchie^+^	1994	Case study	ECT	12 sessions—placement changed at Session 5 (plus fluoxetine: initially 20 mg per day, raised to 60 mg after ECT).	Male outpatient with DPD and MDD (plus comorbid Axis II disorders).	1	24	Overall improvement, including 36 h of lessened DP after Session 8, but DP persisted. ECT stopped after 12 sessions due to mild short-term memory loss.
Simeon et al.	2004	Double-blind RCT	Fluoxetine vs. placebo	10-60 mg per day.	Adults with DPD (*n* = 14/25 in treatment arm had a comorbid depressive disorder).	50	35.7	Fluoxetine did not improve DPDR. Fluoxetine-related improvement in comorbid depression showed no relationship to changes in DPDR.
Psychosis and depression								
Di Michele and Bolino^+^	2004	Case study	Citalopram & Olanzapine	10 mg olanzapine & 10 mg citalopram per day.	Schizophrenia & depression.	1	27	Mr A had been taking olanzapine for four years, adding citalopram to treat a recent depressive episode resolved his hallucinations, low mood, and DPDR after 4–6 weeks.
Fluckiger et al.	2021	Naturalistic uncontrolled study	CBT-informed group therapy (“PLAN-D”)	8× 90-min sessions weekly.	Outpatients with DPDR and: depression (*n* = 4), schizotypal disorder (*n* = 1), bipolar (*n* = 1), CHR (*n* = 6).	8	20.3	All participants who supplied follow-up ratings (*n* = 7) showed reductions in DP six months after baseline. *n* = 4 were below clinical cut off (all were above before). High rates of acceptability.
Anxiety and depression								
Hunter et al.	2005	Open study	CBT for DPD	Mean number of sessions was 13 (SD = 6; range = 4–20).	Outpatients at a DPD clinic: 81% (*n* = 17) had a co-morbid anxiety disorder and/or depression.	21	38	Significant reductions in DP and significant improvement in anxiety and depression scores from baseline to 6-month follow-up.
Preve et al.^+^	2011	Case study	Venlafaxine	112.5 mg per day.	Outpatient with MDD, panic disorder, agoraphobia, and DPDR.	1	21	After 6 months, DP and panic symptoms had remitted (result on depression not stated).
Ratliff and Kerski^+^	1995	Case study	Fluoxetine	20 mg per day (plus 1.5 mg alprazolam – later reduced to 0.25 mg).	Outpatient with panic attacks, depression, and DPDR.	1	36	Adding fluoxetine resulted in ‘remarkable alleviation’ of DP within 2–3 months. Stayed on this combination for 2 years: complete remission of panic, no DPDR, ‘marked decrease’ in depression.
Sierra et al.	2006	Observational	Lamotrigine	209.8 mg per day (average) (range 25-600 mg).	Outpatients of a DPD clinic who had been prescribed lamotrigine over the past 2 years.	32	37.1	Depression, anxiety, and DPDR scores all significantly reduced after treatment.
Zwerenz et al.	2017	RCT	Transdiagnostic affect-focused psychodynamic web-based self-help intervention.	10 weeks of access with guidance.	“Psychosomatic inpatients.”	69	40	The intervention produced a significant decrease in depression and anxiety symptoms, but not for DPDR.
Hunter et al.	2023	Open study	CBT for DPD	Mean of 18.1 sessions. Mean of 17.3 months in therapy	Outpatients at a DPD clinic: 77.8% (*n* = 28) had a co-morbid anxiety disorder and/or depression.	36	-	Clinically significant reductions in DPDR, depression, and anxiety over the treatment period.

+ denotes grey literature. CBT = cognitive-behavioral therapy; CHR = clinically high risk (of psychosis); DP = depersonalisation; DPD = depersonalisation disorder; DPDR = transdiagnostic depersonalisation-derealisation; DR = derealisation; ECT = electro-convulsive therapy; MDD = major depressive disorder; OCD = obsessive compulsive disorder; RCT = randomised controlled trial; rTMS = repetitive transcranial magnetic stimulation; TAU = treatment as usual; VR = virtual reality.

**Table 2:** Summarising the studies pertaining to the third research question for the DPDR in psychosis search.

Author	Year	Type of study	Participants	Mean age	Key DPDR-psychosis finding
Results finding that DPDR and psychosis arise from a shared common cause					
Abel et al.	2003	Experimental	8 healthy adult males.	28.8	Reduced limbic system activity (as induced by ketamine) may be implicated in both DPDR & psychosis.
Biswas et al.	2014	Observational	59 patients with recent-onset psychosis caused by chloroquine (anti-malaria drug).	31.3	“Marked stressors” may be implicated in both DPDR & psychosis.
Hu et al.	2000	Observational	225 patients with psychosis vs. unaffected siblings vs. their parents.	40.5	Val158 allele of COMT gene may be implicated in both DPDR and psychosis.
Maczewska and Barclay^+^	2014	Experimental	30 “good sleepers.”	-	Sleep deprivation may be implicated in both DPDR and psychosis.
Pokorny et al.	2016	Experimental	36 healthy adults.	24.4	5-HT1A receptors may be implicated in both DPDR and complex visual hallucinations.
Schäfer et al.	2012	Observational	145 inpatients with psychosis.	34.0	Childhood sexual abuse may be implicated in both DPDR and psychosis.
Vollenweider et al.	1999	Experimental	7 healthy adult males.	27.0	Increased D2 dopamine binding in the striatum (as induced by psilocybin) may be implicated in DR and psychotic-like experiences.
Results suggesting that DPDR may cause or mediate the causes of psychosis					
Černis et al.	2014	Observational	50 patients with persecutory delusions.	40.4	DP partially mediates the relationship between worry and paranoia.
Dorahy et al.	2023	Observational	45 patients with schizophrenia or schizoaffective disorder.	43.5	DP significantly influenced the circumstantiality and distractibility of formal thought disorder, and the intensity of distress and level of metaphysical interpretation in voice hearing.
Escudero-Pérez et al.	2016	Observational	55 inpatient referrals, scoring 3 or above on hallucinations scale of the PANSS.	38.0	DP was the only significant variable in a regression analysis predicting severity of voice-hearing.
Humpston et al.	2016	Observational	215 healthy adults.	27.1	DP was distinct from, and predicted levels of, delusional ideation and anomalous perceptual experiences.
Kilcommons and Morrison	2005	Observational	32 patients with schizophrenia spectrum disorders.	34.5	DP was a significant predictor of hallucinations.
Morrison and Petersen	2003	Observational	64 undergraduate students and warehouse operatives.	21.0	DP was a significant predictor of predisposition to visual hallucinations.
Peña-Falcón et al.	2018	Observational	167 nonclinical participants.	31.2	DP partially explained the relationship between participants’ sleep quality and hallucination proneness.
Perona-Garcelán et al.	2008	Observational	17 patients with psychosis with auditory hallucinations, vs. 16 patients with psychosis recovered from auditory hallucinations, vs. 18 patients with psychosis who never had auditory hallucinations, vs. 17 non-clinical participants.	38.7	DP was a predictor of hallucinations.
Perona-Garcelán et al.	2011	Observational	59 patients with psychosis.	38.4	DP partially explained the relationship between self-focused attention and experiencing hallucinations.
Perona-Garcelán et al.	2012a	Observational	27 patients with schizophrenia with hallucinations, vs. 20 patients with schizophrenia with delusions but no hallucinations, vs. 28 people recovered from schizophrenia (no symptoms), vs. 22 clinical (no psychosis) control group, vs. 27 non-clinical control group.	37.9	DP was a predictor of hallucinations.
Perona-Garcelán et al.	2013	Observational	318 nonclinical participants with high, medium, and low hallucination proneness.	21.4	DP was a predictor of hallucination proneness.
Therman et al.	2014	Observational (Longitudinal)	731 adolescent psychiatric patients presenting for the first time in 2003–2008.	16.4	DP at first presentation to mental health services was the biggest predictor of whether that person would be admitted to hospital with psychosis in future.
Tschoeke et al.	2022	Observational	118 inpatients with non-psychotic diagnoses.	30.7	DPDR predicted psychoticism, but not paranoia.
Wearne et al.	2018	Observational	69 private practice patients with a diagnosis of PTSD with dissociation.	42.2	DPDR predicted extent of hearing voices (over and above the age at which someone experienced trauma).
Results suggesting psychosis may cause DPDR					
Wright et al.	2020	Observational	58 First Episode Psychosis patients vs. 72 non-clinical control participants.	21.2 vs. 25.7	In clinical group, anomalous perceptual experiences fully mediated relationship between anomalous self-experiences (DP) and delusional beliefs.
Results regarding mediation of the relationship between trauma and psychosis by DPDR					
Bellido-Zanin et al.	2018	Observational	472 university students.	25.5	DP fully mediates the relationship between memory of adverse childhood experiences and hallucination proneness.
Bloomfield et al.	2021	Meta-analysis	24,793 clinical & non-clinical participants.	-	DP partially mediates the relationship between childhood trauma and the experience of hallucinations in adulthood.
Cole et al.	2016	Observational	200 university students.	20.0	DP does not mediate the relationship between childhood maltreatment and hallucination proneness, or between childhood maltreatment and delusional ideation.
Nesbit et al.	2022	Observational	99 inpatients with schizophrenia spectrum disorders or dissociative identity disorder, and outpatients with auditory hallucinations.	49.5	DP is not involved in the relationship between childhood trauma and hallucinations of any sensory modality.
O’Neill et al.	2021	Observational	269 female trauma survivors.	32.1	DP partially explained the relationship between childhood sexual abuse and psychotic-like experiences. It fully explained the relationship between adulthood sexual abuse and psychotic-like experiences.
Perona-Garcelán et al.	2012b	Observational	71 patients with psychosis.	39.1	DP partially explained the relationship between childhood trauma and hallucinations (but not between childhood trauma and delusions).
Perona-Garcelán et al.	2014	Observational	318 nonclinical participants with high vs. low hallucination proneness.	21.4	DP partially explained the relationship between childhood trauma and hallucination proneness.
Results outlining a possible mechanism of DPDR in the context of psychosis					
Freeman et al.	2013	Experimental	67 patients with persecutory delusions.	41.9	An experimental worry manipulation (increase, decrease, or neutral condition) produced significant differences in DP scores.
Johnson^+^	2021	Observational	20 adults with schizophrenia and schizoaffective disorder.	34.4	Anomalous self experiences (ASE) positively predicted DP, but trauma did not. Trauma did not moderate the interaction between ASE and DP.
Wright et al.	2020	Observational	58 First Episode Psychosis patients vs. 72 non-clinical control participants.	21.2 vs. 25.7	In clinical group, DP was predicted by auditory perceptual bias, even after controlling for auditory perceptual sensitivity.

+ denotes grey literature. DP = depersonalisation; DPDR = transdiagnostic depersonalisation-derealisation; DR = derealisation; PANSS = The Positive and Negative Syndrome Scale (Kay et al., 1987); PTSD = post-traumatic stress disorder.

#### 4.1.2 Do any treatments already exist for DPDR in the context of psychosis?

Overall, there was not enough evidence to point towards an effective treatment targeting DPDR in the context of psychosis.

Only one study explicitly approached DPDR as a transdiagnostic target for psychosis (i.e., treating DPDR in order to improve psychosis) (Farrelly et al., 2023), and it showed promising results. However, this was a small feasibility randomised controlled trial. The other treatment studies (Table 1) were single case studies, and two open studies (Rault et al., 2022; Romain et al., 1996). Thus, it is unlikely that any of the included studies were adequately powered to test treatment efficacy, and very few included a follow-up ssessment.

In three reports, the intervention ameliorated psychotic symptoms, but not DPDR (Romain et al., 1996; Narita et al., 2018; Morikawa et al., 1998). However, it is interesting to note that in studies where DPDR did improve, psychotic symptoms also improved (Farrelly et al., 2023; Di Michele and Bolino, 2004; Piedfort-Marin, 2019; Richa et al., 2009; Yoshimura et al., 2020). This is consistent with a hypothesis that transdiagnostic DPDR may be a treatment target for psychosis, but the evidence is far from conclusive.

It is not only the quality and level of the available evidence that must be considered when drawing inferences from the data, but also its generalisability. The heterogeneity of the case studies in terms of participants’ medical comorbidities, the range of interventions applied, and of the reporting of outcomes make it impossible to synthesise the available evidence or to extrapolate findings. The restricted age range represented in studies further limits their generalisability: all studies’ mean ages fell between 20 and 43 years, excluding a significant proportion of the lifespan.

It was noted that studies focusing on treatment of DPDR in psychosis were somewhat clustered geographically (study location was determined by author affiliations. Where this was inconclusive, we used the country from which participants were recruited.). Only one study was from Africa (Cameroon) (Eyoum et al., 2021), and three from Asia (all Japan) (Narita et al., 2018; Morikawa et al., 1998; Yoshimura et al., 2020) – the rest were from Europe. None of the included studies recruited participants from the rest of Africa or Asia Pacific, nor included populations from the Americas or Middle East. The results were also clustered in time: the majority were published in the last 5 years, suggesting relatively recent interest in this topic.

#### 4.1.3 What are the plausible mechanisms of action for DPDR in the context of psychosis?

These results (Table 2) showed clearly that there is a robust and replicable association between psychosis and DPDR, particularly between DPDR and hallucinations or hallucination proneness, which was the focus of the majority of the studies in this section.

Most evidence proposed that DPDR predicted psychosis, or mediated the effect of another variable on psychosis, giving a strong indication that DPDR is a feasible transdiagnostic target in psychosis. However, these studies were almost exclusively cross-sectional observational analyses of cross-sectional data, meaning that it is not possible to confirm the direction of causation. The exception was a prospective longitudinal observational study which supported this direction of causality (Therman et al., 2014).

Within this complexity, the LEAP noted the impact that age of onset and duration of untreated DPDR may have had on these findings, and for understanding the timeframe for causality. For example, Cole et al. (2016) and Bellido-Zanin et al. (2018) both present cross-sectional data from large university student participant groups. However, the former found no mediation by depersonalisation of the relationship between childhood maltreatment and hallucination proneness, whilst the latter – with an average age five years higher – found full mediation by depersonalisation of the relationship between memory of adverse childhood experiences and hallucination proneness. Many other factors may explain these differences, including the specific constructs of ‘maltreatment’ versus ‘memory of adverse experiences’, culture, and measurement factors – but without an understanding of the age of onset of DPDR, the implications of untreated DPDR, and the sequence of events in the causal process from DPDR to psychosis, these cannot be ruled out as explaining the contrasting findings between two similar groups of different ages. These factors were not explored by any of the included results and represent a significant gap in the literature. Further, such observations again highlight that the majority of included studies omitted children, adolescents, and adults over the age of fifty.

Four of the five experimental studies focused on shared causes of both DPDR and psychosis. Much of this evidence suggests DPDR and psychosis may have common biological underpinnings, but is limited by small sample size and assumptions that experimentally-induced DPDR and psychotic symptoms (i.e., via ketamine or psilocybin) are neurocognitively identical to those presenting clinically. Nevertheless, these results may provide a starting point for future genetic, neurocognitive, and pharmacological exploration.

Trauma, particularly childhood abuse and maltreatment, also emerged as an important theme in these results, and was frequently considered as a possible cause of both DPDR and psychotic symptoms. It is beyond the scope of the current review to outline the numerous theories linking trauma to dissociative and psychotic experiences in order to situate our findings within these. It is important to note, though, that all three (dissociation, trauma, psychosis) are broad constructs, within which greater specificity (e.g., DPDR, childhood physical abuse, paranoia) can be reached. Thus, the results presented here pertain only to studies where DPDR was specified, and prohibits a broader understanding of how different types of transdiagnostic dissociation may relate to specific psychotic symptoms and trauma histories. Relatedly, the studies included here focused largely on childhood, as opposed to later-life, trauma.

Three studies explicitly addressed possible mechanisms of DPDR in the context of psychosis (Freeman et al., 2013; Johnson, 2021; Wright et al., 2020). Of these, only one was experimental (Freeman et al., 2013), and demonstrated that manipulating levels of worry in participants with persecutory delusions resulted in corresponding changes in depersonalisation. Thus, worry may be a feasible focus of treatment development for DPDR in the context of psychosis. Indeed, anxiety management strategies formed part of the aforementioned intervention (Farrelly et al., 2023).

### 4.2 DPDR in anxiety

#### 4.2.1 What is the state of the evidence-base for DPDR as a transdiagnostic target for anxiety?

After de-duplication, a similar number of results (*n* = 1,357; Figure 1) were found for the anxiety search as for psychosis. Again, the majority did not concern transdiagnostic DPDR and anxiety disorders (*n* = 889), a further 69 considered DPDR as a side effect of medication only, 99 did not consider the relationship between DPDR and anxiety, and 109 provided correlational results only. A similar number as in the psychosis search (*n* = 138) were considered not sufficiently empirical to be included in this review.

Despite the similarities between the evidence-base for DPDR in anxiety and DPDR in psychosis (Figure 1), we were surprised to note that the distinction between a “symptom” or “experience” on a continuum and a categorical ‘diagnosis’ or ‘disorder’ was more important for the anxiety than the psychosis search. More studies had to be excluded from our results in this search because they approached DPDR as its own diagnostic disorder (DPD). Relatedly, for some studies, it was unclear whether anxiety was conceptualised as a disorder, or as a state or trait experience along a spectrum of severity. There was also a particular challenge when reviewing panic disorder studies, where DPDR was often considered along a continuum, but as a symptom of panic attacks, rather than as its own concept.

Ultimately, 14 treatment studies (8 focusing on anxiety alone, and 6 in combination with depression; Table 1) and 14 mechanistic studies (Table 3) were included. All mechanistic studies were observational. Trauma was a focus in a similar proportion of results (*n* = 3) as for psychosis. The mechanistic studies offered some avenues for future treatment development, including four studies that elucidated mechanisms of DPDR in the context of anxiety.

**Table 3:** Summarising the studies pertaining to the third research question for the DPDR in anxiety search.

Author	Year	Type of study	Participants	Mean age	Key DPDR-Anxiety finding
Results finding that DPDR and anxiety arise from a shared common cause					
Majohr et al.	2011	Observational	95 adults with panic disorder	35.2	Alexithymia (“difficulty in identifying feelings”) predicted DPDR and predicted whether someone was in the panic disorder or non-clinical control group.
Results suggesting that DPDR may cause or mediate the causes of anxiety					
Bridges-Curry et al.	2022	Observational (Longitudinal)	775 participants of the LONGSCAN data collection project (age 16 and age 17 wave data).	-	Age-16 DPDR predicted age-18 anxiety, even after controlling for age-16 anxiety.
Katerndahl	2000	Observational	97 adults meeting DSM-III criteria for panic attacks.	39.6	DP during a panic attack was the only significant predictor of developing agoraphobia.
O’Rourke and Egan	2023	Observational	313 undergraduate students.	22.3	DP was a significant predictor of anxiety. DP mediated the relationship between attachment avoidance and anxiety; and attachment anxiety and anxiety.
Pozza et al.	2016	Observational	60 adults with OCD.	37.2	Appraisal of DP as life threatening was the only significant predictor of ““immediate”“(< 1 year) onset of phobic avoidance after a panic attack.
Schlax et al.	2020	Observational (Longitudinal)	13,182 participants of the Gutenberg Health Study.	54.8	OCD severity was predicted by a combination of higher ‘inferential confusion’ and DPDR severity.
Results suggesting anxiety may cause or mediate the causes of DPDR					
Glaesmer et al.	2013	Observational	693 general population respondents born before 1946.	72.2	Anxiety fully mediated the relationship between World War II involvement/experience and current DPDR.
Mendoza et al.	2011	Observational	104 adults with panic disorder.	37.5	Severity of panic disorder predicted DP.
Results regarding mediation of the relationship between trauma and anxiety by DPDR					
Ford et al.	2018	Observational	809 youth in a short-term juvenile detention centre.	16.1	DPDR significantly mediated the relationship between poly-victimisation and anxiety/depression score on screening measure.
Ó Laoide et al.	2018	Observational	761 university students.	24.5	DP was a significant mediator of the relationship between emotional maltreatment and anxiety.
Santoro et al.	2023	Observational	333 non-clinical adults.	25.6	DPDR partially mediated the relationship between traumatic experiences (emotional abuse and emotional neglect—not physical abuse) and obsessive-compulsive symptoms.
Results outlining a possible mechanism of DPDR in the context of anxiety					
Cook and Newins	2021	Observational	572 psychology students.	20.58	Emotion regulation difficulties (specifically “difficulties controlling impulsive behavior” and ‘lack of emotional clarity’) moderated the relationship between social anxiety and DPDR.
Geerts et al.^+^	2015	Observational	One inpatient with treatment-resistant depression (and comorbid generalised anxiety disorder & hyperthyroidism).	31	High-frequency rTMS over the dorsolateral prefrontal cortex created DPDR symptoms that took months to resolve.
Ó Laoide et al.	2018	Observational	761 university students.	24.5	Emotional maltreatment, attachment-related anxiety, attitudes towards emotional expression, and not getting 7-9 h sleep were significant predictors of DP.
Vannikov-Lugassi et al.	2021	Observational (Longitudinal)	98 outpatients with depression, an anxiety disorder, and/or OCD (& 49 non-clinical participants).	38.9	Rumination predicted DPDR in the following month. DPDR did not predict rumination in the following month.

+ denotes grey literature. DPDR = transdiagnostic depersonalisation-derealisation; DP = depersonalisation; DSM-III = Diagnostic and Statistical Manual of Mental Disorders (3rd ed.); LONGSCAN = Longitudinal Studies on Child Abuse and Neglect (Runyan et al., 2014); OCD = obsessive compulsive disorder; rTMS = repetitive transcranial magnetic stimulation.

Regarding the generalisability of the current evidence-base, it was noted that nearly all studies were from the USA or Europe, and most concerned individuals or groups with (mean) ages between 20 and 40. The exception to this was Pegna et al. (1999), presenting a case study of a 12-year-old girl: this was the only treatment study across all three searches with an age below 20. The time-clustering seen in the psychosis search was not as evident in the results of this search.

#### 4.2.2 Do any treatments already exist for DPDR in the context of anxiety?

Again, there were optimistic signs that good treatment effects are possible for DPDR in this context. However, it was not possible to conclude from these findings whether DPDR is a feasible treatment target in the context of anxiety.

Of the eight treatment studies focusing solely on anxiety (Table 1), four focused specifically on panic disorder. The remaining four focused on social anxiety, generalised anxiety disorder, obsessive compulsive disorder, and one study of depersonalisation disorder where participants with a range of comorbid anxiety disorders were analysed as a sub-set of the data. In total, four results were single case studies, one was a case series of three patients, and two were RCTs. Only two – the case series of *n* = 3 (McKay and Moretz, 2008); and one of the RCTs (Schweden et al., 2016) – tested a psychological intervention, the rest reported on pharmacological interventions. All focused on short-term treatment effects.

Of the further six treatment studies that explored DPDR in the context of comorbid anxiety and depression, two were single case studies (both reporting remission following pharmacological interventions). There were also three open studies (two psychological interventions, one pharmacological) showing good effect, and one RCT of an internet-based psychological intervention that did not improve DPDR, despite improving depression and anxiety (Zwerenz et al., 2017).

Overall, the treatment studies for anxiety showed mixed results: whilst some found that their intervention improved both DPDR and anxiety symptoms concurrently, there was a pattern in pharmacological reports that clinicians’ first choice medication improved anxiety, but not DPDR, requiring further prescribing (Preve et al., 2013; Ratliff and Kerski, 1995; Uguz and Sahingoz, 2014; Weber, 2020). It is not known whether the medication that improved DPDR would also have improved anxiety symptoms if trialled as a first line option. This is an important hypothesis to test since both included RCTs that aimed to target anxiety (Schweden et al., 2016; Mavissakalian, 1996) imply that doing so reduces DPDR, suggesting that that anxiety is a feasible treatment target for DPDR. The reverse – transdiagnostic DPDR as a target for anxiety – does not find support in these results. However, again, only two studies (Hunter et al., 2023; Simeon et al., 2004) specifically targeted DPDR, Simeon et al. (2004) show that improvements in DPDR and anxiety are linked, and Hunter et al.’s psychological intervention also explicitly included therapeutic techniques for anxiety and depression. This means that a robust exploration of DPDR as a transdiagnostic treatment target for anxiety has yet to be undertaken.

#### 4.2.3 What are the plausible mechanisms of action for DPDR in the context of anxiety?

Mechanistic studies focusing on the relationship between anxiety and DPDR (Table 3) were more likely than the treatment studies to suggest that DPDR is a feasible target for anxiety, this apparent contradiction perhaps reflecting the LEAP’s lived experience of a reciprocal relationship between the two. However, at an individual level, the majority of studies used a cross-sectional observational design, and the LEAP noted that these tended to assume a simplistic unidirectional relationship between constructs, rather than being capable of modelling a bidirectional relationship situated within a complex broader context. For example, the LEAP described lived experience of nuance between somatic and cognitive elements of anxiety, and of these having different relationships to DPDR. This was not reflected in the results – perhaps due to the focus on diagnostic categories of anxiety. Further, the LEAP suggested that to better understand DPDR in relation to anxiety, future research should consider how, and under what conditions, they may reinforce one another, and be reinforced by associated experiences, such as stigma, multimorbidity, and meta-cognitive processes. Whilst the LEAP highlighted that there were no studies included in the current review that addressed stigma, from a clinical psychology standpoint, it may be helpful to consider whether fear of judgement related to stigma may already be addressed in the context of cognitive therapy for social anxiety, where beliefs about other people’s negative evaluations are typically a focus of intervention strategies (Clark and Wells, 1995). Indeed, this may explain the result of Schweden et al. (2016), above.

Factors highlighted by these results that could be assessed in future research to determine whether they are feasible mechanisms of DPDR in this context included: alexithymia (as important to both DPDR and anxiety; Majohr et al., 2011); rumination (Vannikov-Lugassi et al., 2021); and emotion regulation (Cook and Newins, 2021), attachment style, and attitudes towards emotional expression (Laoide et al., 2018). The rumination result echoes the above discussion of the relationships between worry, DPDR, and psychosis. Suggestions that difficulties identifying or regulating, or having negative attitudes towards affect are important in dissociative experiences are also supported elsewhere (Černis et al., 2022; Evren et al., 2012; McGuinness et al., 2025).

### 4.3 DPDR in depression

#### 4.3.1 State of the evidence

Fewer results were found for the depression search than for anxiety or psychosis (*n* = 991; Figure 1). There were, however, similar proportions of findings that were excluded on the basis of not concerning transdiagnostic DPDR and depressive disorders (*n* = 705), considering DPDR only as a medication side effect (*n* = 40), not considering the relationship between DPDR and depression (*n* = 60), presenting only a correlational understanding of the relationship (*n* = 69), or being insufficiently empirical (*n* = 76). Additionally, six were excluded because they conceptualised DPDR as a symptom of depression, operationalised using a single item in the Hamilton Depression Rating Scale (HAM-D; Hamilton, 1960), which was judged not to be an adequate characterisation of transdiagnostic DPDR.

Research into DPDR in the context of depression alone (rather than comorbid with anxiety, as above) was notably sparse. Six treatment studies focused on depression, and a further eight addressed depression alongside psychosis (*n* = 2) or anxiety (*n* = 6) (Table 1). Only ten mechanistic studies were found that addressed the issue of transdiagnostic DPDR in the context of a depressive disorder (Table 4). Of these, only two were unique to the depression search (Ghaemi Kerahrodi et al., 2022; Yoshimasu et al., 2006), the rest also relevant to understanding of DPDR in the context of anxiety. The scarcity of depression-specific studies was highlighted as an important limitation by the LEAP, since there is arguable overlap between the experience of DPDR and depression (for example, numbed mood, thoughts of suicide, and links to deliberate self-harm). Indeed, the LEAP relayed anecdotal evidence from their own experience and others’ that DPDR is often confused with depression by clinicians.

**Table 4:** Summarising the studies pertaining to the third research question for the DPDR in depression search.

Author	Year	Type of study	Participants	Mean age	Key DPDR-Depression finding
Results suggesting that DPDR may cause or mediate the causes of depression					
Bridges-Curry et al.	2022	Observational (longitudinal)	775 participants of the LONGSCAN data collection project (age 16 & age 17 wave data).	-	Age-16 DPDR did not predict age-18 depression.
Ghaemi Kerahrodi et al.^+^	2022	Observational (longitudinal)	10,422 participants of the Gutenberg Health Study.	-	Participants with DP at baseline had higher odds ratio of significant depression at 5-year follow up.
O’Rourke and Egan	2023	Observational	313 university students.	22.3	DP was a significant predictor of depression. DP mediated the relationship between ‘attachment avoidance’ and depression; and ‘attachment anxiety’ and depression.
Schlax et al.	2020	Observational (longitudinal)	13,182 participants of the Gutenberg Health Study.	54.8	DPDR predicted depression symptoms 2.5 years later, even after controlling for baseline anxiety, depression, and medical conditions.
Yoshimasu et al.	2006	Observational	199 outpatients with depression.	40.3	DP was significant predictor of suicidal ideation in men. DR was a significant predictor in women.
Results suggesting depression may cause or mediate the causes of DPDR					
Glaesmer et al.	2013	Observational	693 general population respondents born before 1946.	72.2	Depression fully mediated the relationship between World War II involvement/experience and DPDR.
Results regarding mediation of the relationship between trauma and depression by DPDR					
Ford et al.	2018	Observational	809 youth in a short-term juvenile detention centre.	16.1	DPDR significantly mediated the relationship between poly-victimisation and anxiety/depression score on a screening measure.
Ó Laoide et al.	2018	Observational	761 university students.	24.5	DP was a significant mediator of the relationship between emotional maltreatment and depression.
Results outlining a possible mechanism of DPDR in the context of depression					
Geerts et al.^+^	2015	Observational	One inpatient with treatment-resistant depression (and comorbid generalised anxiety disorder & hyperthyroidism)	31	High-frequency rTMS over the dorsolateral prefrontal cortex created DPDR symptoms that took months to resolve.
Vannikov-Lugassi et al.	2021	Observational (Longitudinal)	98 outpatients with depression, an anxiety disorder, and/or OCD (& 49 non-clinical participants).	38.9	Rumination predicted DPDR in the following month. DPDR did not predict rumination in the following month.

+ denotes grey literature. DP = depersonalisation; DPDR = transdiagnostic depersonalisation-derealisation; DR = derealisation; LONGSCAN = Longitudinal Studies on Child Abuse and Neglect (Runyan et al., 2014); OCD = obsessive compulsive disorder; rTMS = repetitive transcranial stimulation.

The significant overlap between the anxiety and depression search results means that the geographical locations and cultural contexts of the depression studies are similar to those discussed above.

#### 4.3.2 Do any treatments already exist for DPDR in the context of depression?

Results addressing treatment in the context of co-occurring DPDR and depression (only) were almost exclusively case studies: five presented case studies of six patients, and the aforementioned RCT (Simeon et al., 2004) was included here due to its sub-analysis of participants with comorbid depressive disorders (Table 1).

Overall, these results are inconclusive with regards to the viability of transdiagnostic DPDR as a treatment target for depression, and generally found that alleviation of depressive symptoms was a more reliable treatment outcome than improvement of DPDR.

#### 4.3.3 What are the plausible mechanisms of action for DPDR in the context of depression?

Few studies addressed the relationship between transdiagnostic DPDR and depressive disorders (*n* = 10; Table 4). More so than for anxiety, it was often unclear whether depression was being measured as a diagnostic entity, or along a continuum of low mood. This is particularly relevant given the number of large cohort studies in this section of the review (*n* = 4) (Ghaemi Kerahrodi et al., 2022; Bridges-Curry et al., 2022; Glaesmer et al., 2013; Schlax et al., 2020). Within these studies, it also appeared that DPDR was typically only assessed using the two-item version of the Cambridge Depersonalisation Scale (CDS-2; Michal et al., 2011), or the DPDR items of a trauma scale, and perhaps was not intended to be the focus of the study when the data collection was originally devised.

In terms of understanding the relationship between depressive disorders and transdiagnostic DPDR, it was difficult to find a clear pattern within the results. Whilst one study found no association (Bridges-Curry et al., 2022), four found some evidence that DPDR might cause or mediate levels of depressive symptomatology (Ghaemi Kerahrodi et al., 2022; Yoshimasu et al., 2006; Schlax et al., 2020; O’Rourke and Egan, 2023), and one found that DPDR depression fully mediated DPDR (Glaesmer et al., 2013). Consistent with other sections, two results also identified DPDR as a mediator between trauma and depression (Laoide et al., 2018; Ford et al., 2018).

Only two papers offered insight into factors that may be important in future treatment development. As above, Vannikov-Lugassi et al. (2021) highlighted the role of rumination; and Geerts et al. (2015) suggests that the left dorsolateral prefrontal cortex (DLPFC) may be a brain region of interest. In this study, a patient with treatment-resistant depression (and comorbid generalised anxiety disorder) received stimulation via high-frequency repetitive transcranial magnetic stimulation (r-TMS) that resulted in long-lasting DPDR symptoms. This is particularly interesting given that findings of *improvements* in DPDR with rTMS stimulation of the left DLPFC (Karris et al., 2017). In fact, in this patient – who also had treatment-resistant depression – reduction of DPDR was achieved only after switching from the right DLPFC to the left.

## 5 Discussion

This scoping review aimed to provide an overview of the evidence available for DPDR as a transdiagnostic treatment target, and to synthesise its key findings. It has revealed an evidence-base that is largely under-developed and, in places, surprisingly sparse. Only a minority of studies retrieved through our searches considered the dynamics of the relationship between DPDR and anxiety, depression, or psychosis, fewer still sought to directly target DPDR in these contexts, and no established effective treatment options were identified.

More specifically, these results indicate that DPDR is highly likely to be a viable treatment target in the context of psychosis, although confirmation via adequately powered studies with appropriate methodology is required. The findings suggest that DPDR and anxiety disorders are tightly linked, perhaps sharing important cognitive maintenance processes, such as fear of social evaluation and maladaptive beliefs about affect. However, as a result of this close – possibly bidirectional – relationship, the feasibility of DPDR as a treatment target in this context is obscured, and this hypothesis has yet to be robustly tested. In depressive disorders, the small number of heterogenous results meant it was impossible to identify any consistent findings. However, as in all sections of this review, there were many indications of potentially fruitful avenues for future research.

### 5.1 What is the state of the evidence-base for DPDR as a transdiagnostic target for anxiety, depression, and psychosis?

As noted above, the evidence-base for DPDR as a transdiagnostic target is in the early stages of development. There was only one study that specifically targeted DPDR as an intervention for anxiety, depression or psychosis (Farrelly et al., 2023). However, there were promising signs that the interaction between psychosis and DPDR has been attracting increasing research interest in recent years.

The current evidence-base is lacking in representation. No research appeared to include gender or sexual minorities, and most results either did not report ethnicity within their participant group, or else recruited predominantly White participants. This is perhaps a reflection of the geographical clustering of the included results, which largely came from Western Europe and North America. The LEAP also noted that neurodiversity was completely missing in these results, as were younger (child and adolescent), and older (age fifty and above) age groups. Omission of older age groups is a concern, especially given how important feelings of disconnection and unreality could feasibly be in the developmental context of this stage of life. Omission of younger age groups is also a concern: the LEAP highlighted their own experiences of problematic DPDR occurring sometimes before the age of ten, but this information is missing within the evidence-base.

### 5.2 Do any treatments already exist for DPDR in the context of anxiety, depression, or psychosis?

There was not enough evidence to point towards a clear and efficacious treatment for transdiagnostic DPDR in any of these three contexts. However, whilst startlingly little treatment evidence exists for transdiagnostic DPDR, there were clear signs of promise for future treatment development efforts. At present, pharmacological, psychosocial, and technological (virtual reality, repetitive transcranial magnetic stimulation) interventions all appear to be viable avenues for future treatment development.

In particular, some pharmacological case reports demonstrated promising improvements in both transdiagnostic DPDR and the accompanying psychiatric diagnosis, but these require more rigorous testing before they can be recommended. The methodology of such reports was often unclear, with some medications given in combination, some lacking clarity about discontinuation and subsequent ‘wash-out’ periods, and some that may reasonably be expected to have been prescribed alongside other medications not stated (for example, in the cases of physical health comorbidities).

Arguably, the most persuasive evidence in the current review was a psychological therapy targeting important cognitive processes within DPDR (e.g., rumination) (Farrelly et al., 2023), but this feasibility study was not statistically powered to test treatment efficacy. Although the most developed interventions in this review were psychological therapies, these, too, require further development. Such interventions were either highly targeted, in early stages of development, and need statistically-powered tests of efficacy (e.g., Farrelly et al., 2023); or else were better established, but still need to be understood in terms of their method of action regarding transdiagnostic DPDR [e.g., (Schweden et al., 2016)].

Surprisingly, whilst trauma was often implicated as a common cause of DPDR, anxiety, depression, and psychosis, no psychological intervention studies appeared to test trauma-focused therapy. This is a notable gap in the literature, but may perhaps be explained by the specification of DPDR as the dissociative subtype under scrutiny in this review. For example, work elsewhere has addressed dissociative experiences more generally than this, to good effect [e.g., in psychosis (Geerts et al., 2015; Varese et al., 2021)].

### 5.3 What are the plausible mechanisms of action for DPDR in these contexts (that could be the focus of treatment development in future)?

A strength of the current evidence-base is the wealth of practical research questions it poses regarding the mechanisms of action for transdiagnostic DPDR across these three diagnostic categories.

In the psychosis section of the review, there were neurological and genetic findings that may be worthy of further exploration, and promising developments in psychological interventions. The latter appear to incorporate strategies targeting plausible mechanisms of DPDR identified elsewhere in this section of the review: namely, rumination.

Rumination was also implicated in the context of anxiety and depressive disorders, suggesting it may be a transdiagnostic mechanism for DPDR. This lends further support to the cognitive-behavioral model of DPD (Hunter et al., 2003), which suggests that the disorder is maintained by health anxiety-like rumination and monitoring of DPDR experiences in response to catastrophic cognitive appraisals.

In the anxiety section of the review, affect-related factors such as alexithymia, attitudes towards affect, and emotion regulation, also appeared to be important – echoing recent findings in related areas of dissociation (Černis et al., 2022; McGuinness et al., 2025).

Besides rumination, and one neurobiological finding, there were no immediately obvious mechanisms of action implicated for DPDR in the depression section of the review. Perhaps confirming whether some of the above suggestions also hold true in this context would be a logical first step in this area.

### 5.4 Clinical implications

On the weight of the current evidence-base, it is not possible to recommend an effective treatment strategy for transdiagnostic DPDR.

The dearth of clinical treatment studies may be a reflection of the relative lack of familiarity clinicians have with DPDR and dissociation, as highlighted elsewhere (Brand, 2016), and therefore potentially points towards a training need amongst mental healthcare providers.

In the absence of evidence-based guidance, service user and lived experience involvement is ever more crucial in care provision and planning. Specifically, people with DPDR should be involved in shared decision-making regarding treatment options, and in decisions about what ‘recovery’ looks like. Unfortunately, these aspects of clinical practice were not typically made explicit in the reports included in this review.

It is also important for clinicians to note that it was common for intervention studies to find that a treatment alleviated either DPDR or the diagnostic context being considered, but not both. This, and the lived experience of the phenomenology of transdiagnostic DPDR highlights that whilst DPDR may have a close relationship to anxiety, depression, and psychosis symptoms it is nevertheless a separable experience.

### 5.5 Research implications

The vast majority of studies in this review used observational data, meaning that where a direction of effect was assumed or hypothesized, this could not be verified. Thus, more studies designed to detect causation – and preferably those that can also test for reciprocal relationships – are sorely needed and should be a research priority in this field. In contrast, there is an abundance of research reporting correlations between transdiagnostic DPDR and anxiety, depression, and psychosis. We recommend that the focus now turns to understanding the finer detail of this association.

In the field of psychosis, future research should take care to consider the full range of psychotic symptoms, since most findings in this section of the review concerned hallucinations or hallucination proneness. As above, more work is needed to establish the extent of overlap between depressive and dissociative symptoms, and whether any causal relationships exist between them.

Regarding mechanisms of transdiagnostic DPDR, we note that biological mechanisms were under-represented in the included studies and constitute another area of research opportunity. The LEAP also raised that the importance of age of onset and impact of duration of untreated illness are left unaddressed by this review. Little is known about how these factors influence the severity of transdiagnostic DPDR, and consequently, its diagnostic context. Similarly, it is unclear what factors may moderate illness or recovery.

As noted above, greater diversity is required within the evidence-base to ensure that it is representative. This is particularly important with regards to neurodiversity, which clinicians note often overlaps significantly in clinical presentations of dissociation (R. Andrew, *personal communication,* 21 March 2023).

Again, given the relative lack of research into dissociative experiences, people with lived experience of DPDR should be actively involved in setting research priorities, and in the planning and delivery of these.

### 5.6 Strengths and limitations

This scoping review represents an attempt to fairly apply a robust framework to a complex and extremely varied set of search results. It is likely that the application of this framework has led to the omission of potentially helpful results for two reasons. First, the lack of consistency and precision of conceptualisation and measurement of key constructs meant that some high-quality work was excluded because the authors did not distinguish which type(s) of dissociative experience were being considered. This is a similar difficulty as that reported in a meta-analysis of research in this area (Pilton et al., 2015). This is important, since different subtypes of dissociative experience can show contrasting findings [e.g., absorption versus depersonalisation (Cole et al., 2016)] – and thus further attention should be given to what precise construct is being measured. Second, the focus on transdiagnostic DPDR meant that studies exploring Depersonalisation-Derealisation Disorder (DPD; i.e., a diagnostic entity in its own right) were excluded. These may have included useful information about treatment, as it is feasible that treatments for DPD may be viable in transdiagnostic contexts.

In an attempt to standardise this search, we have also omitted diagnoses that could arguably have been included: most notably bipolar disorder, where depression is a key clinical feature. This decision was made *a priori* to reduce the scope of the review and reduce the complexity of the key constructs, but this may be one reason why the depression search produced relatively few results. Relatedly, including more general and specific terms for positive and negative psychotic symptoms (e.g., cognitive disorganisation) may also helped widened the search.

A further limitation of our methodology is the lack of geographic diversity in our LEAP: despite our efforts to advertise via global networks, only one member was located outside of the UK. Thus, the above findings are lacking in lived experience insights from diverse cultural perspectives.

It is a strength of this review that the search results were not limited to English language, and that grey literature was included. However, given that our search terms were in English, it is likely that this restricted the search: perhaps explaining the geographical clustering and lack of diversity of the final results, and the absence of findings regarding culture-bound syndromes and cultural expressions of DPDR. More concerted efforts to explore the scope of the current evidence-base in these respects are required.

## 6 Conclusion

Transdiagnostic DPDR does appear to be a feasible treatment target in at least anxiety and psychosis, but further research is required to more fully characterise the relationship between DPDR and depressive disorders. In all contexts, research designs allowing causal inference would be valuable additions to the evidence-base. Treatment development work is also required, since targeted treatment options for transdiagnostic DPDR are scarce, and none have yet been tested for treatment efficacy.

Given the relative lack of familiarity with DPDR within mainstream mental health services, and since it is difficult to draw clinically meaningful conclusions from the current evidence-base, we recommend that lived experience experts should be actively involved in setting the research priorities in this area, and that clinicians should use shared decision making when drawing up care plans for DPDR.

Nearly a decade on, we reprise the call for “innovative thinking and research” (Şar, 2014) to advance this “neglected” area of mental health. To his characterisation of this field as an opportunity to make significant progress, we add the consideration that the implications of such work may well be felt beyond the confines of dissociative disorders, and could have repercussions transdiagnostically.

## Data Availability

The original contributions presented in the study are included in the article/Supplementary material, further inquiries can be directed to the corresponding author

## Footnotes

^1^ https://doi.org/10.17605/OSF.IO/UFBKN

## The Transdiagnostic DPDR Project Lived Experience Advisory Panel

Louise Chandler, L. Corrigan, Nanette Lee, Sara Metz, and Judah Njoroge.
