# Supplementary material for: Depersonalisation-derealisation as a transdiagnostic treatment target: a scoping review of the evidence in anxiety, depression, and psychosis
Source: Front Psychol. 2025 Apr 16;16:1531633. doi: 10.3389/fpsyg.2025.1531633 (PMC12042760; doi:10.3389/fpsyg.2025.1531633)
Supplement: Supplementary file 1 [file Supplementary_file_1.docx]

## Appendix A

Audio-visual summaries of this project are available from: <https://osf.io/ufbkn/files/osfstorage>

Below is a ‘plain English’ summary, authored by JP and edited by The Transdiagnostic DPDR Project Lived Experience Advisory Panel:

This project looked at how depersonalisation and derealisation (DPDR) symptoms can accompany three other experiences: psychosis, anxiety, and depression. After reviewing global academic papers from the last 30 years, we tried to identify patterns in the findings to see if they might improve our current understanding of DPDR and highlight areas for future study that have not previously been considered. We involved lived experience advisors throughout: two assisting in managing the project; holding regular meetings with our Lived Experience Advisory Panel (LEAP) to interpret the results; and contracting artists with DPDR to create a short video and infographic to help circulate the findings online.

Multiple papers suggested treating DPDR might be important in psychosis and could lead to improvements in psychotic symptoms as a result. The intensity of DPDR also reliably predicted the level of certain psychosis symptoms, though not all. However, treatment papers were largely limited in their sample size (many were individual case studies) and non-treatment papers mainly focussed on people aged 20 to 45. Further, one of the larger studies that found no correlation involved only university students. Given DPDR onset most commonly occurs during teenage years, we cannot be sure whether this limited age range affected the findings or not.

Many papers from the anxiety search had to be excluded as they dealt with DPDR as a standalone disorder rather than a transdiagnostic (cross-cutting) experience – which was the focus of this project. From the remaining results, many had an emphasis on panic disorders – where DPDR was considered a secondary symptom of panic attacks rather than its own concept – and others were unclear whether they were viewing anxiety as a disorder or an everyday emotional experience. Overall, it was difficult to draw firm conclusions about the direction of influence between anxiety and DPDR, possibly because they are often a joint experience for many people. But some papers did highlight promising potential avenues that future research into DPDR treatment could explore.

The depression search found significantly fewer results, as most papers seemed to combine depression with anxiety and considered them together. As such, identifying any significant patterns indicating a possible relationship between DPDR and depression was not possible. However, whilst some studies specifically highlighted that they found no association, others did loosely suggest DPDR being linked with more severe or complex depression, rather than mild. Our LEAP suggested one reason for the lack of results might be an overlap between depression and DPDR symptoms, leading to DPDR not being identified specifically in many cases.

Overall, our analysis showed that possible relationships between these three diagnoses and DPDR still need verifying. And, whilst important trends were identified and some papers did show optimistic results, there wasn’t any evidence of clear or effective treatments for DPDR. Much more diverse and inclusive research is needed in this area to understand the causes and effects of DPDR and develop new treatments. Such research should involve larger sample sizes of participants; across broader age ranges; and with increased guidance from lived experience experts.

## Appendix B

References for final results included in the scoping review:

Abel, K. M., Allin, M. P. G., Kucharska-Pietura, K., David, A., Andrew, C., Williams, S., Brammer, M. J., & Phillips, M. L. (2003). Ketamine alters neural processing of facial emotion recognition in healthy men: An fMRI study. *NeuroReport*, *14*(3), 387–391. <https://doi.org/10.1097/00001756-200303030-00018>

Belli, H., Akbudak, M., Ural, C., & Aslaner, D. (2014). A case of depersonalization with treatment-resistant depression successfully treated with sertraline-lamotrigine combination. *West Indian Medical Journal*, *63*(1), 115. <https://doi.org/10.7727/wimj.2012.303>

Bellido-Zanin, G., Perona-Garcelán, S., Senín-Calderón, C., López-Jiménez, A. M., Ruiz-Veguilla, M., & Rodríguez-Testal, J. F. (2018). Childhood memories of threatening experiences and submissiveness and its relationship to hallucination proneness and ideas of reference: The mediating role of dissociation. *Scandinavian Journal of Psychology*, *59*(4), 407–413. <https://doi.org/10.1111/sjop.12455>

Biswas, P. S., Sen, D., & Majumdar, R. (2014). Psychosis following chloroquine ingestion: A 10-year comparative study from a malaria-hyperendemic district of India. *General Hospital Psychiatry*, *36*(2), 181–186. <https://doi.org/10.1016/j.genhosppsych.2013.07.012>

Bloomfield, M. A. P., Chang, T., Woodl, M. J., Lyons, L. M., Cheng, Z., Bauer-Staeb, C., Hobbs, C., Bracke, S., Kennerley, H., Isham, L., Brewin, C., Billings, J., Greene, T., & Lewis, G. (2021). Psychological processes mediating the association between developmental trauma and specific psychotic symptoms in adults: A systematic review and meta-analysis. *World Psychiatry*, *20*(1), 107–123. <https://doi.org/10.1002/wps.20841>

Bridges-Curry, Z., Christian, C., & Levinson, C. A. (2022). Network Analysis of PTSD Symptoms in a Sample of Polyvictimized Youth. *Journal of Trauma and Dissociation*, *23*(4), 401–415. <https://doi.org/10.1080/15299732.2021.1989115>

Černis, E., Dunn, G., Startup, H., Kingdon, D., Wingham, G., Pugh, K., Cordwell, J., Mander, H., & Freeman, D. (2014). Depersonalization in Patients With Persecutory Delusions. *Journal of Nervous & Mental Disease*, *202*(10), 752–758. <https://doi.org/10.1097/NMD.0000000000000185>

Cole, C. L., Newman-Taylor, K., & Kennedy, F. (2016). Dissociation mediates the relationship between childhood maltreatment and subclinical psychosis. *Journal of Trauma and Dissociation*, *17*(5), 577–592. <https://doi.org/10.1080/15299732.2016.1172537>

Cook, M. A., & Newins, A. R. (2021). Social anxiety and dissociation: The moderating role of emotion regulation. *Motivation and Emotion*, *45*(3), 345–353. <https://doi.org/10.1007/s11031-021-09875-5>

Di Michele, V., & Bolino, F. (2004). Adjunctive citalopram is effective on hallucinations and depersonalization symptoms: A case report. *European Psychiatry*, *19*(3), 185. <https://doi.org/10.1016/j.eurpsy.2003.07.013>

Dorahy, M. J., Nesbit, A., Palmer, R., Wiltshire, B., Cording, J. R., Hanna, D., Seager, L., & Middleton, W. (2023). A comparison between auditory hallucinations, interpretation of voices, and formal thought disorder in dissociative identity disorder and schizophrenia spectrum disorders. *Journal of Clinical Psychology*, *79*(9), 2009–2022. <https://doi.org/10.1002/jclp.23522>

Escudero-Perez, S., Leon-Palacios, M. G., Ubeda-Gomez, J., Barros-Albarran, M. D., Lopez-Jimenez, A. M., & Perona-Garcelan, S. (2016). Dissociation and mindfulness in patients with auditory verbal hallucinations. *Journal of Trauma and Dissociation*, *17*(3), 294–306. <https://doi.org/10.1080/15299732.2015.1085480>

Eyoum, C., Mbenda, N. K., Kontchou, R. T., Belle, S. N. E., & Njiengwe, E. (2021). Role of psychomotricity in the management of body image disorders in schizophrenia: A case report. *Pan African Medical Journal*, *40*, 184. <https://doi.org/10.11604/pamj.2021.40.184.27107>

Farrelly, S., Peters, E., Azis, M., David, A. S., & Hunter, E. C. M. (2023). A brief CBT intervention for depersonalisation-derealisation disorder in psychosis: Results from a feasibility randomised controlled trial. *Journal of Behavior Therapy and Experimental Psychiatry*, 101911. <https://doi.org/10.1016/j.jbtep.2023.101911>

Flückiger, R., Schmidt, S. J., Michel, C., Kindler, J., & Kaess, M. (2021). Introducing a Group Therapy Program (PLAN D) for Young Outpatients with Derealization and Depersonalization: A Pilot Study. *Psychopathology*, *55*(1), 62–68. <https://doi.org/10.1159/000520008>

Ford, J. D., Charak, R., Modrowski, C. A., & Kerig, P. K. (2018). PTSD and dissociation symptoms as mediators of the relationship between polyvictimization and psychosocial and behavioral problems among justice-involved adolescents. *Journal of Trauma and Dissociation*, *19*(3), 325–346. <https://doi.org/10.1080/15299732.2018.1441354>

Freeman, D., Startup, H., Dunn, G., Cernis, E., Wingham, G., Pugh, K., Cordwell, J., & Kingdon, D. (2013). The interaction of affective with psychotic processes: A test of the effects of worrying on working memory, jumping to conclusions, and anomalies of experience in patients with persecutory delusions. *Journal of Psychiatric Research*, *47*(12), 1837–1842. <https://doi.org/10.1016/j.jpsychires.2013.06.016>

Geerts, P.-J., Lemmens, G. M. D., & Baeken, C. (2015). The occurrence of depersonalization symptoms after accelerated HF-rTMS of the left DLPFC in a patient with treatment-resistant depression: A case report. *Brain Stimulation*, *8*(3), 681–682. <https://doi.org/10.1016/j.brs.2015.02.010>

Ghaemi Kerahrodi, J., Beutel, M., Wiltink, J., Wild, P., Munzel, T., Lackner, K., Konig, J., Pfeiffer, N., Nagler, M., & Michal, M. (2022). The depersonalization/derealization subtype of depression—Longitudinal results from the Gutenberg Health Study. *Journal of Psychosomatic Research*, *157*, 110852. <https://doi.org/10.1016/j.jpsychores.2022.110852>

Glaesmer, H., Michal, M., Beutel, M. E., & Brahler, E. (2013). The association between war-related traumatic experiences and depersonalization, anxiety and depression symptomatologies in Germany’s World War II generation. *Kriegsbezogene traumatische Erfahrungen, Depersonalisation, Angst- und Depressionssymptomatil in der Weltkrieg-ll-Generation in Deutschland.*, *7*(3), 230–238.

Hu, X., Zhou, R., Chen, C., Wang, Y., & Zhou, Z. (2000). Linkage or association analysis between Val158Met polymorphism of catechol-O-methyltransferase gene and schizophrenia in Chinese Han. *Chinese Mental Health Journal*, *14*(6), 375–378.

Humpston, C. S., Walsh, E., Oakley, D. A., Mehta, M. A., Bell, V., & Deeley, Q. (2016). The relationship between different types of dissociation and psychosis-like experiences in a non-clinical sample. *Consciousness and Cognition*, *41*, 83–92. <https://doi.org/10.1016/j.concog.2016.02.009>

Hunter, E. C. M., Baker, D., Phillips, M. L., Sierra, M., & David, A. S. (2005). Cognitive-behaviour therapy for depersonalisation disorder: An open study. *Behaviour Research and Therapy*, *43*(9), 1121–1130. <https://doi.org/10.1016/j.brat.2004.08.003>

Hunter, E. C. M., Wong, C. L. M., Gafoor, R., Lewis, G., & David, A. S. (2023). Cognitive Behaviour Therapy (CBT) for Depersonalization Derealization Disorder (DDD): A self-controlled cross-over study of waiting list vs. active treatment. *Cognitive Behaviour Therapy*, *52*(6), 672–685. <https://doi.org/10.1080/16506073.2023.2255744>

Johnson, G. E. (2021). Examination of trauma, depersonalization, and schizophrenia through the ipseity disturbance model. *Dissertation Abstracts International: Section B: The Sciences and Engineering*, *82*(9-B).

Karris, B. C., Capobianco, M., Wei, X., & Ross, L. (2017). Treatment of depersonalization disorder with repetitive transcranial magnetic stimulation. *Journal of Psychiatric Practice*, *23*(2), 141–144. <https://doi.org/10.1097/PRA.0000000000000215>

Katerndahl, D. A. (2000). Predictors of the development of phobic avoidance. *Journal of Clinical Psychiatry*, *61*(8), 618–623. <https://doi.org/10.4088/JCP.v61n0813a>

Kilcommons, A. M., & Morrison, A. P. (2005). Relationships between trauma and psychosis: An exploration of cognitive and dissociative factors. *Acta Psychiatrica Scandinavica*, *112*(5), 351–359. <https://doi.org/10.1111/j.1600-0447.2005.00623.x>

Maczewska K.B. & Barclay N.L. (2014). The effects of 18 hours of sustained wakefulness on changes in paranoid and delusional beliefs in good sleepers. *Sleep*, *37*(SUPPL. 1), A90.

Majohr, K.-L., Leenen, K., Grabe, H. J., Jenewein, J., Nuñez, D. G., & Rufer, M. (2011). Alexithymia and Its relationship to dissociation in patients with panic disorder. *Journal of Nervous and Mental Disease*, *199*(10), 773–777. <https://doi.org/10.1097/NMD.0b013e31822fcbfb>

Mavissakalian, M. R. (1996). Phenomenology of panic attacks: Responsiveness of individual symptoms to imipramine. *Journal of Clinical Psychopharmacology*, *16*(3), 233–237. <https://doi.org/10.1097/00004714-199606000-00007>

McKay, D., & Moretz, M. W. (2008). Interoceptive Cue Exposure for Depersonalization: A Case Series. *Cognitive and Behavioral Practice*, *15*(4), 435–439. <https://doi.org/10.1016/j.cbpra.2008.05.002>

Mendoza, L., Navinés, R., Crippa, J. A., Fagundo, A. B., Gutierrez, F., Nardi, A. E., Bulbena, A., Valdés, M., & Martín-Santos, R. (2011). Depersonalization and personality in panic disorder. *Comprehensive Psychiatry*, *52*(4), 413–419. <https://doi.org/10.1016/j.comppsych.2010.09.002>

Morikawa, M., Iida, J., & Kishimoto, T. (1998). A case of schizophrenia with pituitary gigantism: Concomitant bromocriptine therapy with neuroleptics. *Journal of Nara Medical Association*, *49*(5), 412–419.

Morrison, A. P., & Petersen, T. (2003). Trauma, Metacognition And Predisposition To Hallucinations In Non-Patients. *Behavioural and Cognitive Psychotherapy*, *31*(3), 235–246. <https://doi.org/10.1017/S1352465803003011>

Narita, Z., Satake, N., Sato, W., & Takano, H. (2018). Possible effects of electroconvulsive therapy on refractory psychosis in primary progressive multiple sclerosis: A case report. *Neuropsychopharmacology Reports*, *38*(2), 92–94. <https://doi.org/10.1002/npr2.12014>

Nesbit, A., Dorahy, M. J., Palmer, R., Middleton, W., Seager, L., & Hanna, D. (2022). Dissociation as a Mediator Between Childhood Abuse and Hallucinations: An Exploratory Investigation Using Dissociative Identity Disorder and Schizophrenia Spectrum Disorders. *Journal of Trauma and Dissociation*, *23*(5), 521–538. <https://doi.org/10.1080/15299732.2022.2064579>

Ó Laoide, A., Egan, J., & Osborn, K. (2018). What was once essential, may become detrimental: The mediating role of depersonalization in the relationship between childhood emotional maltreatment and psychological distress in adults. *Journal of Trauma and Dissociation*, *19*(5), 514–534. <https://doi.org/10.1080/15299732.2017.1402398>

O’Neill, T., Maguire, A., & Shevlin, M. (2021). Sexual trauma in childhood and adulthood as predictors of psychotic-like experiences: The mediating role of dissociation. *Child Abuse Review*, *30*(5), 431–443. <https://doi.org/10.1002/car.2705>

Ordas, D. M., & Ritchie, E. C. (1994). Treatment of depersonalization disorder and associated depression with electroconvulsive therapy. *The Journal of Neuropsychiatry and Clinical Neurosciences*, *6*(1), 67–69. <https://doi.org/10.1176/jnp.6.1.67-a>

O’Rourke, N., & Egan, J. (2023). The Effects of Emotion Regulation on Physical and Psychological Wellbeing in University Students: The Role of Depersonalization and Attachment Style. *Journal of Trauma and Dissociation*, *24*(3), 426–444. <https://doi.org/10.1080/15299732.2023.2181473>

Pegna, C., Perri, A., & Lenti, C. (1999). Panic disorder or temporal lobe epilepsy: A diagnostic problem in an adolescent girl. *European Child and Adolescent Psychiatry*, *8*(3), 237–239. <https://doi.org/10.1007/s007870050134>

Peña-Falcón, M. R., Pascualena-Nagore, C., & Perona-Garcelán, S. (2018). Unusual sleep experiences and dissociation as mediators between sleep quality and proneness to hallucinations in a nonclinical population sample: A preliminary study. *Cognitive Neuropsychiatry*, *23*(2), 88–102. <https://doi.org/10.1080/13546805.2018.1439733>

Perona-Garcelán, S., Cuevas-Yust, C., García-Montes, J. M., Pérez-Álvarez, M., Ductor-Recuerda, M. J., Salas-Azcona, R., Gómez-Gómez, M. T., & Rodríguez-Martín, B. (2008). Relationship between self-focused attention and dissociation in patients with and without auditory hallucinations. *Journal of Nervous and Mental Disease*, *196*(3), 190–197. <https://doi.org/10.1097/NMD.0b013e318165c7c1>

Perona-Garcelán, S., Carrascoso-López, F., García-Montes, J. M., Vallina-Fernández, O., Pérez-Álvarez, M., Ductor-Recuerda, M. J., Salas-Azcona, R., Cuevas-Yust, C., & Gómez-Gómez, M. T. (2011). Depersonalization as a mediator in the relationship between self-focused attention and auditory hallucinations. *Journal of Trauma and Dissociation*, *12*(5), 535–548. <https://doi.org/10.1080/15299732.2011.602181>

Perona-Garcelán, S., García-Montes, J. M., Ductor-Recuerda, M. J., Vallina-Fernández, O., Cuevas-Yust, C., Pérez-Álvarez, M., Salas-Azcona, R., & Gómez-Gómez, M. T. (2012a). Relationship of metacognition, absorption, and depersonalization in patients with auditory hallucinations. *The British Journal of Clinical Psychology*, *51*(1), 100–118. <https://doi.org/10.1111/j.2044-8260.2011.02015.x>

Perona-Garcelán, S., Carrascoso-López, F., García-Montes, J. M., Ductor-Recuerda, M. J., López-Jiménez, A. M., Vallina-Fernández, O., Pérez-Álvarez, M., & Gómez-Gómez, M. T. (2012b). Dissociative experiences as mediators between childhood trauma and auditory hallucinations. *Journal of Traumatic Stress*, *25*(3), 323–329. Embase. <https://doi.org/10.1002/jts.21693>

Perona-Garcelán, S., García-Montes, J. M., Rodríguez-Testal, J. F., Ruiz-Veguilla, M., Benítez-Hernández, M. D. M., López-Jiménez, A. M., Arias-Velarde, M. A., Ductor-Recuerda, M. J., Gómez-Gómez, M. T., & Pérez-Álvarez, M. (2013). Relationship of absorption, depersonalisation, and selffocused attention in subjects with and without hallucination proneness. *Cognitive Neuropsychiatry*, *18*(5), 422–436. <https://doi.org/10.1080/13546805.2012.728133>

Perona-Garcelán, S., García-Montes, J. M., Rodríguez-Testal, J. F., López-Jiménez, A. M., Ruiz‐Veguilla, M., Ductor-Recuerda, M. J., Benítez-Hernández, M. M., Arias-Velarde, M. A., Gómez-Gómez, M. T., & Pérez-Álvarez, M. (2014). Relationship Between Childhood Trauma, Mindfulness, and Dissociation in Subjects With and Without Hallucination Proneness. *Journal of Trauma and Dissociation*, *15*(1), 35–51. <https://doi.org/10.1080/15299732.2013.821433>

Piedfort-Marin, O. (2019). The theory of the structural dissociation of the personality for the conceptualization and treatment of dissociative psychosis: Two case studies. *Annales Medico-Psychologiques*, *177*(8), 788–795. <https://doi.org/10.1016/j.amp.2018.04.013>

Pokorny, T., Preller, K. H., Kraehenmann, R., & Vollenweider, F. X. (2016). Modulatory effect of the 5-HT1A agonist buspirone and the mixed non-hallucinogenic 5-HT1A/2A agonist ergotamine on psilocybin-induced psychedelic experience. *European Neuropsychopharmacology*, *26*(4), 756–766. <https://doi.org/10.1016/j.euroneuro.2016.01.005>

Pozza, A., Torniai, S., & Dettore, D. (2016). Inferential confusion moderates the effects of dissociative experiences on OCD symptoms severity in a clinical sample with obsessive-compulsive disorder. *Clinical Neuropsychiatry*, *13*(6), 108–114.

Preve, M., Mula, M., Cassano, G. B., & Pini, S. (2011). Venlafaxine in somatopsychic and autopsychic depersonalization. *Progress in Neuro-Psychopharmacology & Biological Psychiatry*, *35*(8), 1808–1809. <https://doi.org/10.1016/j.pnpbp.2011.06.011>

Preve, M., Mula, M., Favaretto, E., Basaglia, C., & Schwitzer, J. (2013). Ziprasidone augmentation of lamotrigine in treatment depersonalization in bipolar disorder. *European Psychiatry*, *28*(SUPPL. 1).

Ratliff III, N. B., & Kerski, D. (1995). Depersonalization treated with fluoxetine. *American Journal of Psychiatry*, *152*(11), 1689–1690.

Rault, O., Lamothe, H., & Pelissolo, A. (2022). Therapeutic use of virtual reality relaxation in schizophrenia: A pilot study. *Psychiatry Research*, *309*, 114389. <https://doi.org/10.1016/j.psychres.2022.114389>

Richa, S., Bayle, F., & Loo, H. (2009). Schizophrenia and Niemann-Pick disease. *Primary Psychiatry*, *16*(3), 31–32.

Romain, J. L., Dermain, P., Greslé, P., Grignon, S., Moisan, P., Nore, D., Pech, G., Benyaya, J., & Perret, I. (1996). Efficacy of zuclopenthixol acetate on psychotic anxiety assessed during an open study. *Encephale*, *22*(4), 280–286.

Rotaru, T.-Ş., Hîngənescu, A. R., & Alexa, T. (2015). Intervention for chronic dysthimia: A case study on diagnostic uncertainty. *Journal of Psychological and Educational Research*, *23*(2), 90–110.

Santoro, G., Sideli, L., Gugliemucci, F., Terrone, G., & Schimmenti, A. (2023). Traumatic Experiences and Obsessive-Compulsive Symptoms: The Mediating Role of Dissociation. *The Journal of Nervous and Mental Disease*, *211*(7), 543. <https://doi.org/10.1097/NMD.0000000000001649>

Schäfer, I., Fisher, H. L., Aderhold, V., Huber, B., Hoffmann-Langer, L., Golks, D., Karow, A., Ross, C., Read, J., & Harfst, T. (2012). Dissociative symptoms in patients with schizophrenia: Relationships with childhood trauma and psychotic symptoms. *Comprehensive Psychiatry*, *53*(4), 364–371. <https://doi.org/10.1016/j.comppsych.2011.05.010>

Schlax, J., Wiltink, J., Beutel, M. E., Münzel, T., Pfeiffer, N., Wild, P., Blettner, M., Ghaemi Kerahrodi, J., & Michal, M. (2020). Symptoms of depersonalization/derealization are independent risk factors for the development or persistence of psychological distress in the general population: Results from the Gutenberg health study. *Journal of Affective Disorders*, *273*, 41–47. <https://doi.org/10.1016/j.jad.2020.04.018>

Schweden, T. L. K., Pittig, A., Bräuer, D., Klumbies, E., Kirschbaum, C., & Hoyer, J. (2016). Reduction of depersonalization during social stress through cognitive therapy for social anxiety disorder: A randomized controlled trial. *Journal of Anxiety Disorders*, *43*, 99–105. <https://doi.org/10.1016/j.janxdis.2016.09.005>

Sierra, M., Baker, D., Medford, N., Lawrence, E., Patel, M., Phillips, M. L., & David, A. S. (2006). Lamotrigine as an add-on treatment for depersonalization disorder: A retrospective study of 32 cases. *Clinical Neuropharmacology*, *29*(5), 253–258. Scopus. <https://doi.org/10.1097/01.WNF.0000228368.17970.DA>

Simeon, D., Guralnik, O., Schmeidler, J., & Knutelska, M. (2004). Fluoxetine therapy in depersonalisation disorder: Randomised controlled trial. *British Journal of Psychiatry*, *185*(JULY), 31–36. <https://doi.org/10.1192/bjp.185.1.31>

Therman, S., Lindgren, M., Manninen, M., Loewy, R. L., Huttunen, M. O., Cannon, T. D., & Suvisaari, J. (2014). Predicting psychosis and psychiatric hospital care among adolescent psychiatric patients with the Prodromal Questionnaire. *Schizophrenia Research*, *158*(1–3), 7–10. <https://doi.org/10.1016/j.schres.2014.06.031>

Tschoeke, S., Flammer, E., Bichescu-Burian, D., & Steinert, T. (2022). The Association between Type of Dissociation and Psychotic Experiences in a Non-Psychotic Inpatient Sample. *Journal of Trauma and Dissociation*, *23*(5), 504–520. <https://doi.org/10.1080/15299732.2022.2064576>

Uguz, F., & Sahingoz, M. (2014). Aripiprazole in depersonalization disorder comorbid with major depression and obsessive-compulsive disorder: 3 cases. *Clinical Neuropharmacology*, *37*(4), 125–127. <https://doi.org/10.1097/WNF.0000000000000036>

Vannikov-Lugassi, M., Shalev, H., & Soffer-Dudek, N. (2021). From brooding to detachment: Rumination longitudinally predicts an increase in depersonalization and derealisation. *Psychology and Psychotherapy: Theory, Research and Practice*, *94*(S2), 321–338. <https://doi.org/10.1111/papt.12279>

Vollenweider, F. X., Vontobel, P., Hell, D., & Leenders, K. L. (1999). 5-HT modulation of dopamine release in basal ganglia in psilocybin-induced psychosis in man—A PET study with [11C]raclopride. *Neuropsychopharmacology : Official Publication of the American College of Neuropsychopharmacology*, *20*(5), 424–433. <https://doi.org/10.1016/S0893-133X(98)00108-0>

Wearne, D., Curtis, G., Choy, W., Magtengaard, R., Samuel, M., & Melvill-Smith, P. (2018). Trauma-intrusive hallucinations and the dissociative state. *BJPsych Open*, *4*(5), 385–388. <https://doi.org/10.1192/bjo.2018.52>

Weber, S. R. (2020). Use of mixed amphetamine salts in a patient with depersonalization/derealization disorder. *Innovations in Clinical Neuroscience*, *17*(1–3), 45–48.

Wright, A., Nelson, B., Fowler, D., & Greenwood, K. (2020). Perceptual biases and metacognition and their association with anomalous self experiences in first episode psychosis. *Consciousness and Cognition: An International Journal*, *77*, 4(May), 1-26. <https://doi.org/10.1016/j.concog.2019.102847>

Yoshimasu, K., Sugahara, H., Tokunaga, S., Akamine, M., Kondo, T., Fujisawa, K., Miyashita, K., & Kubo, C. (2006). Gender differences in psychiatric symptoms related to suicidal ideation in Japanese patients with depression. *Psychiatry and Clinical Neurosciences*, *60*(5), 563–569. <https://doi.org/10.1111/j.1440-1819.2006.01559.x>

Yoshimura, R., Ikenouchi, A., & Konishi, Y. (2020). Improvement of persistent cenesthopathy and depersonalization after treatment with brexpiprazole in schizophrenia. *Clinical Neuropsychopharmacology and Therapeutics*, *11*, 43–44. <https://doi.org/10.5234/cnpt.11.43>

Zwerenz, R., Becker, J., Johansson, R., Frederick, R. J., Andersson, G., & Beutel, M. E. (2017). Transdiagnostic, psychodynamic web-based self-help intervention following inpatient psychotherapy: Results of a feasibility study and randomized controlled trial. *JMIR Mental Health*, *4*(4). <https://doi.org/10.2196/mental.7889>
